# Antiproliferative Properties of Triterpenoids by ECIS Method—A New Promising Approach in Anticancer Studies?

**DOI:** 10.3390/molecules27103150

**Published:** 2022-05-14

**Authors:** Anna Hordyjewska, Monika Prendecka-Wróbel, Łukasz Kurach, Anna Horecka, Anna Olszewska, Dominika Pigoń-Zając, Teresa Małecka-Massalska, Jacek Kurzepa

**Affiliations:** 1 Chair and Department of Medical Chemistry, Medical University of Lublin, 4A Chodzki Str., 20-093 Lublin, Poland; anna.hordyjewska@umlub.pl (A.H.); anna.horecka@umlub.pl (A.H.); jacek.kurzepa@umlub.pl (J.K.); 2 Chair and Department of Human Physiology, Medical University of Lublin, 11 Radziwiłłowska Str., 20-093 Lublin, Poland; monika.prendecka-wrobel@umlub.pl (M.P.-W.); anna.olszewska@umlub.pl (A.O.); dominika.pigon-zajac@umlub.pl (D.P.-Z.); teresa.malecka-massalska@umlub.pl (T.M.-M.); 3 Independent Laboratory of Behavioral Studies, Medical University of Lublin, 4A Chodzki Str., 20-093 Lublin, Poland

**Keywords:** impedance, ECIS, antitumor activity, anticancer properties, betulin, betulinic acid

## Abstract

Electric cell–substrate impedance sensing is an advanced in vitro impedance measuring system which uses alternating current to determine behavior of cells in physiological conditions. In this study, we used the abovementioned method for checking the anticancer activities of betulin and betulinic acid, which are some of the most commonly found triterpenes in nature. In our experiment, the threshold concentrations of betulin required to elicit antiproliferative effects, verified by MTT and LDH release methods, were 7.8 µM for breast cancer (T47D), 9.5 µM for lung carcinoma (A549), and 21.3 µM for normal epithelial cells (Vero). The ECIS results revealed the great potential of betulin and betulinic acid’s antitumor properties and their maintenance of cytotoxic substances to the breast cancer T47D line. Moreover, both substances showed a negligible toxic effect on healthy epithelial cells (Vero). Our investigation showed that the ECIS method is a proper alternative to the currently used assay for testing in vitro anticancer activity of compounds, and that it should thus be introduced in cellular routine research. It is also a valuable tool for live-monitoring changes in the morphology and physiology of cells, which translates into the accurate development of anticancer therapies.

## 1. Introduction

**Figure 1:**
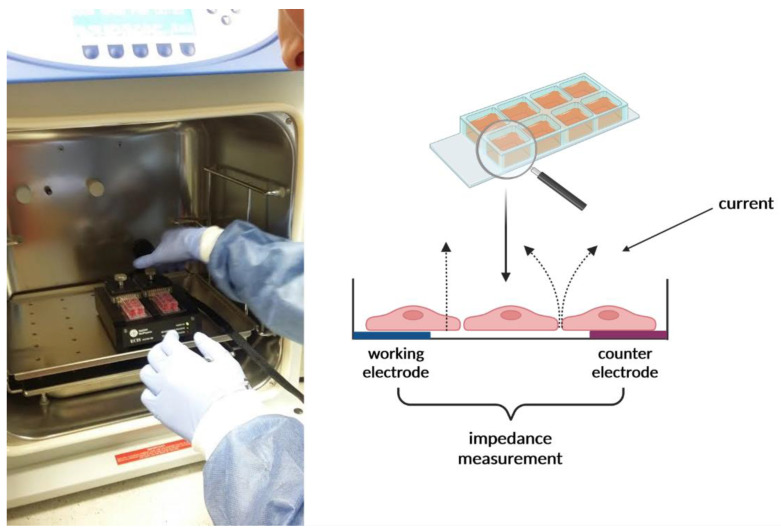
ECIS measuring station in the incubator (photo from the cell culture laboratory, Chair, and Department of Human Physiology, Medical University of Lublin). The scheme was created with BioRender.

Routine analysis carried out on cell lines usually allows for the assessment of one parameter at the same time, such as the number, morphology, and phenotype of cells, as well as the viability and metabolic activity of the cell, the synthesis of intracellular transcription factors, or the release of many regulatory proteins. The impedance method seems to be a promising, non-invasive technique for studying cells and their morphological changes caused by various stimuli. Giaever and Keese were the pioneers of this technique. They developed electric cell–substrate impedance sensing (ECIS) as a continuous monitoring system to study cell behavior using a real-time and label-free method [1,2,3]. These measurements detect changes in the morphology of the cells, which, in turn, are derived from specific metabolic processes. In this system, electrodes are used to apply low alternating current. Those electrodes which measure the voltage change are mounted at the bottom of a standard matrix. When the cells attach to the matrix and electrodes, they act as insulators, increasing impedance. The flow of the current is hindered depending on the number of cells covering the electrode, cells’ shape, and the type of cells attached to the electrode surface. The structure design of the ECIS measuring system includes (Figure 1): two electrodes (one is a small working electrode and the other a large counter electrode on the bottom of the culture plate) connected to the edge of a culture chip—the chip is connected to the lock-in amplifier. The whole setup is placed inside an incubator in steady conditions of 37 °C and under a 5% CO_2_ atmosphere. After cell seeding, cells drift downward and attach to the stratum of the electrode, which then passes the impeded current directly into the bulk electrolyte as the result of anchored plasma membrane intrusion above the electrode surface [1,4,5].

In this system, frequency is a very important factor. At low frequencies (<2000 Hz), the majority of the current flows between the cells. At high frequencies (>40,000 Hz), most of the current flows directly through the isolated cell membranes. The impedance at high frequencies is affected more significantly by the cell membrane, while at low frequencies, the response is more dependent on the space below and between cells [2,4,6].

ECIS is capable of detecting morphology changes in the subnanometer to micrometer range. In ECIS, a small alternating current (I) is applied across the electrode pattern at the bottom of the ECIS arrays (a direct current (DC) cannot be used). This results in a potential (V) across the electrodes which is measured by the ECIS instrument. As cells grow and cover the electrodes, the current is impeded in a manner related to the number of cells covering the electrode, the morphology of the cells, and the nature of the cell attachment. When cells are stimulated to change their function, the accompanying changes in cell morphology alter the impedance. The data generated are impedance versus time [1,7,8].

Ohm’s law is the basis for the measurement of the electrical impedance of biological objects (Formula (1)), which describes the relation between resistance (R), current (I), and voltage (U) in an electrical circuit at a given time (t):

R(t) = U(t)/I(t)(1)

However, it should be noted that within the AC system, current and voltage not only differ in their amplitude but also their phase (φ). In this case, resistance alone is not sufficient to describe these relations. Instead, the complex impedance (Z) or, in most cases, the magnitude of the impedance (|Z|) is used, containing resistance plus reactance (X), which results from AC flow through capacitors and inductors driving the phase shift between voltage and current [9,10].

Thus, in AC systems:

|Z(f)| = √[R^2^ +X(f)^2^] 
φ = arctan (X/R)(2)

Due to the characteristics of cell membrane, when performing impedance measurements on intact cells, the cells form connections with the resistor and the capacitor, in parallel. Here, resistance represents the opposition to current flow, whereas capacitance (C) describes the separation of electric carriers at the insulating bilayer of the cell membrane that causes polarization of the cell. The direct parameters derived from impedance measurements are the resistance and capacitance of cells. The quality and function of the cell barrier are represented by the resistance and, therefore, the resistance towards para- and trans-cellular current flow should be considered. Capacitance provides an overall measure of electrode coverage [9,11,12].

Therefore, the different behavior of the cells after their seeding, adherence, proliferation, and reaction to the substances added to the substrate, as a result, produces a change in impedance. To check whether the ECIS method would be a good method for assessing antiproliferative and anticancer activity, we chose compounds belonging to the triterpene family: betulin and betulinic acid. Betulin (BE; lup-20(29)-ene-3β,28-diol) and betulinic acid (BA; 3β-hydroxy-lup-20(29)-en-28-oic acid) belong to triterpenes and are found in the outer layer of the bark of white birch species such as *Betula alba*, *Betula verrucasa*, or *Betula pendula* [13]. The most important pharmacological activity of these triterpenoids is the inhibition of the development of some chemo-resistant tumors, such as melanoma or gliomas [10,14,15,16,17]. Although BE and BA are very similar in structure, there is a major difference in their cytotoxic activity—BA is less cytotoxic to healthy cells than BE [18]. However, both of them are characterized by a lack of toxicity both in vitro and in vivo within normal cells, and that is the reason why BE and BA are considered potential precursors of many new medicinal preparations [14,19,20].

This study aimed to determine whether the anticancer effects of betulin and betulinic acid on human lung carcinoma (A549) and human breast carcinoma (T47D) lines can be determined by the ECIS method, or only by common tests such as MTT or LDH. We chose such a configuration of cancer cell lines because lung cancer usually metastasizes to the nipple first [21]. For the Vero cell line, non-pathological epithelial cells served as a control for the experiment.

## 2. Results

### 2.1. The MTT Results

To examine whether BE and BA have cytotoxic activity in chosen cell line types, we first assessed their effect on the viability of cell lines with an MTT assay during a 48 h culture period. As shown in Table 1, BE inhibited cell growth in T47D, A549, and Vero cells with an IC_50_ of 7.8, 9.5, and 21.3 µM, respectively. BA, a structure-related derivative of betulin, showed a potent antiproliferative effect with lower IC_50_ values in the same cell lines (5.4, 6.9, and 18.6 µM, respectively), indicating that betulin has a mechanism slightly distinct from BA in the inhibition of cell growth.

### 2.2. The LDH Results

**Figure 2:**
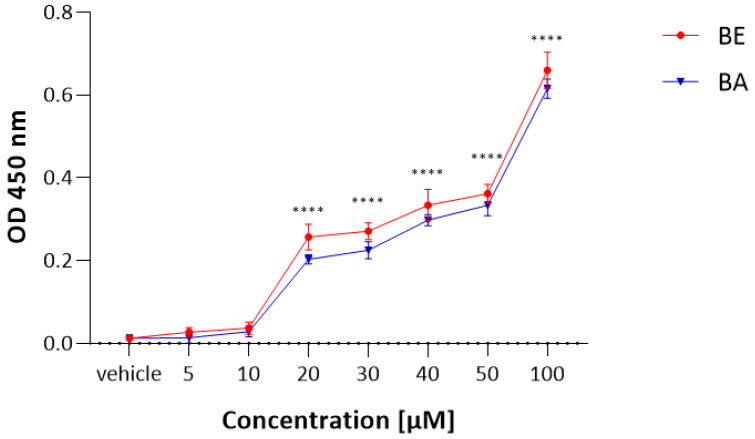
The influence of BE and BA on Vero cell viability measured by LDH assay. Data are shown as means ± SD; **** *p* < 0.0001 vs. vehicle; Dunnett’s test.

To obtain a reliable IC_50_ value of BE and BA towards a Vero cell, we applied another cytotoxicity test but, this time, using a different mechanism of action to MTT. Vero cells were treated with increasing doses of BE and BA, ranging from 1 µM to 100 µM during a 48 h culture period. LDH release was observed after 48 h of BE and BA treatment, and this release was significantly increased with 20 µM of betulin and betulinic acid (Figure 2). The LDH assay revealed that both compounds caused modest cytotoxicity in normal Vero cell culture, consistent with the results obtained from the MTT assay. Accordingly, 20 µM of BA and BE were used in further studies concerning Vero cell lines.

### 2.3. The ECIS Results

**Figure 3:**
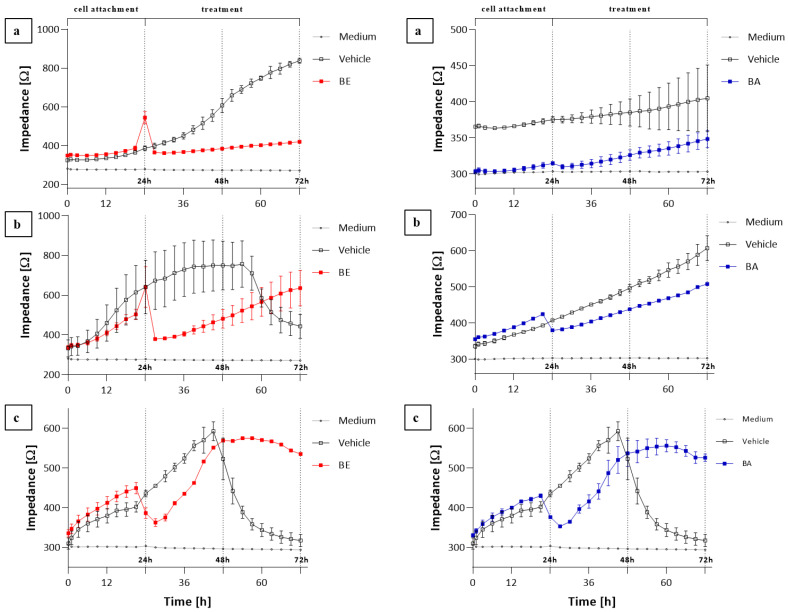
Impedance changes monitoring of the cell line (**a**) A549, (**b**) T47D, (**c**) Vero during 48 h treatment with BE (red line) and BA (blue line). Data are presented as mean value ± SEM.

Using the ECIS system, we monitored selected cell lines (T47D, A549, and Vero) continuously for up to 72 h and noted significant changes in impedance after the administration of appropriately selected concentrations of BE (8, 10, and 20 μM) and BA (5, 7, and 20 µM). Based on the obtained results, it can be noted that both substances affect electrical parameters during cultivation in a different mode (Figure 3). Every cell type has its characteristic adhesion and growth curve that can be manipulated by stimuli such as the chemical structure or concentration of substances in the medium. As made clear at the starting phase (cell attachment), the impedance increased in all cell cultures: A549 (Figure 3a), T47D (Figure 3b), and Vero (Figure 3c) cells, and it grew even further (negative control). After the addition of BE and BA, the impedance increase was insignificant for the A549 line, whereas recorded impedance values of the T47D line treated with BE showed a sharp decrease in impedance values and a slight decrease when treated with BA, followed by a linear increase in values for both cases. A similar trend of impedance value changes was observed for the Vero line.

**Figure 4:**
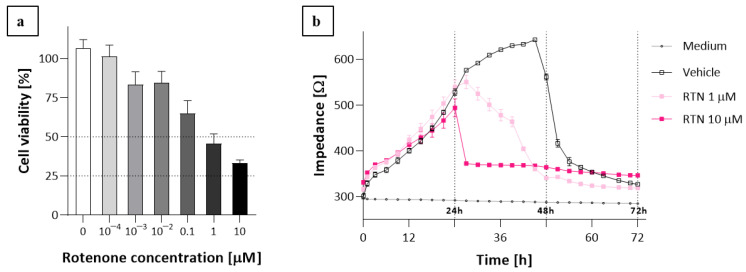
Influence of rotenone on Vero cell line during 48 h treatment assessed by (**a**) MTT assay and monitored by (**b**) ECIS method. Data are presented as mean value ± SEM.

Moreover, to present the influence of a well-known cytotoxic substance on the Vero cell line, rotenone was used. Cell viability was assessed after 48 h of incubation by the MTT assay (Figure 4a); then, two concentrations were used for ECIS monitoring (Figure 4b). The influence of a lower concentration of rotenone was characterized by a slow decrease in impedance, while treatment with a higher concentration resulted in an immediate decrease in impedance.

## 3. Discussion

Explaining the relationship between changes in electrical parameters in cell cultures and the processes influencing its survival seems to be an interesting challenge. No matter how satisfactory this answer might be, in the face of new technical possibilities (such as ECIS) and assuming that changes in the electrical properties of cells precede changes on the biochemical level, it would be very interesting to examine the character and dynamics of these changes. It is possible only with the monitoring of selected electrical parameters, i.e., the impedance, resistance, and capacity of the cell membrane, in real time and after the application of chosen bioactive compounds to the examined cell lines [9,10,11,22].

Drug discovery and screening of bioactive compounds are often performed in cell-based test systems to reduce costs and save time. Traditionally, microscopy, spectrophotometry, and flow cytometry techniques are used to examine cell cultures. The abovementioned methods are considered standard in studies conducted on cell cultures. While these methods may provide insight into the physiological function of every single cell or into pathological changes that could have occurred, they usually require fluorescence, chemiluminescence, or radioactive ways of marking, which lead to cell destruction. The marking process causes the loss of important biological information about living cells [23,24]. ECIS is an innovative and non-invasive method used to monitor cell parameters such as cell membrane capacity, resistance, or impedance in real-time analysis. Additionally, ECIS measurements provide information about temporal changes in the cell–cell contacts which are not available for single-cell observations. Depending on the experimental setup, ECIS measurements show an excellent time resolution, ranging from seconds (for example, micromotion) to minutes. So far, this technique has been successfully used in the study of cell viability, the determination of IC_50_, and the influence of various biological factors on cellular processes [25,26].

Previous studies have reported that BE and BA have an anticancer effect in human lung or breast cancer cell lines. In our experiment, we also confirmed the antiproliferative activity of these compounds on such lines, and our obtained IC_50_ values are in agreement with the literature [17,27,28] on the matter. Comparisons made using IC_50_ (Table 1) showed that the most sensitive cell line to BA and BE was T47D (breast carcinoma). This agrees with the results obtained by other authors [17,29]. Results regarding ECIS are rather similar, but their analysis allows us to observe the accurate influence of BE and BA on cells. Based on impedance measurements in the cultures of the examined cells exposed to BE, significant differences in the impedance values were observed. Likewise, the A549 line was the most sensitive to the action of BE. After BE addition, further cell growth was stopped rapidly compared to the negative control and lasted until the end of incubation, which indicates the antiproliferative effect of the test substance on these cells. If we compare the effect of BE on A549 cells, it can be seen that, unlike with betulin, the effect of betulinic acid on these cells cannot be demonstrated.

In the case of T47D cells (Figure 3b), the effect of BE was slightly different. After substance addition, a gradual but significant drop in impedance and, subsequently, a slow and linear growth was observed. Given that during the experiment the medium was not exchanged, the drop in impedance in the negative control cultures reflects the increase in cell deaths associated with the depletion of nutrients and the accumulation of metabolites. Therefore, the effect of betulin appears to be all the more interesting since, according to the results, it seems to prolong the life of T47D cells. On the other hand, BA had a pronounced and efficiently antiproliferative effect on T47D cells.

The effects of BE and BA on the cells of the Vero line are similar (Figure 3c) to those of T47D cells, although the increase in cell proliferation is greater, demonstrating the tolerance of these cells towards BE and BA, which was also confirmed with other normal lines [14,19,20]. Rotenone—an inhibitor of complex I of the electron transport chain (ETC)—was used as a positive control at two concentrations, which is a good example to illustrate the changes in impedance when a toxicant is used at concentrations higher than the IC50. Rotenone use at the 10 µM level caused an immediate decrease in the impedance value, proving a strong antiproliferative effect which was also confirmed by the MTT test.

The literature surrounding the study suggests that the selective cytotoxic activity of BE and BA may result from their direct influence on the mitochondrial bioenergetics and functioning of the lipid membrane. A well-known feature of neoplastic cells is their intensive proliferation, which means the intensification of their energy changes. Mitochondria lie at the centers of these changes, so the fate of the cell depends on their proper functioning. Although BE and BA are structurally similar, differing only in the substituent at C-17, they may act differently on the same cell type, for example, inducing the release of cytochrome C from the mitochondria in different ways [30]. Aside from this, possible triterpene–lipid interactions should also be considered. Rodrıguez et al. showed that pentacyclic monohydroxytriterpenes affect the dynamics and structural properties of the artificial lipid bilayer dipalmitoylphosphatidylcholine (DPPCB) and that some of the triterpenes, such as α-amyrin, are incorporated into the lipid bilayer at the same high concentrations as cholesterol [31]. The susceptibility of steroid-like compounds to incorporation into the artificial lipid membranes of multilamellar liposomes of DPPCB is strongly dependent on their structure [32,33]. Thus, the hypothesis for the existence of interactions between triterpenes and cellular membrane lipids (their having an ordering or destabilizing influence on the lipid components of the membrane) does not seem unfounded. Few studies focused on the structural similarity of BE and BA with cholesterol [33,34]. It is widely recognized that one of the most important structural functions of cholesterol is modulating the fluidity of lipid membranes. By blending in between the elastic membrane phospholipids, it modifies their interactions, consequently modulating the dynamics of the bilayer [35,36,37]. A similar effect is attributed to BE and BA: it is presumed that, due to their structural similarity to cholesterol, they may exhibit similar properties to the aforementioned cholesterol or to other steroids and thus show a very high affinity for lipid cell membranes. Nevertheless, the effect on the membranes may not be the same. Dubinin et al. reported that BE changes the surface properties of the lipid membrane, facilitating its aggregation or fusion. Furthermore, BE and BE can react with mitochondrial permeability transition (MPT) pores in the mitochondria [38]. Carvalho et al. came to similar conclusions on the effect of BE on the plasticity of lysosomal membranes [39]. Moreover, lipid membranes are mainly made of cardiolipin and have low cholesterol levels. Consequently, their activity may be disturbed by an excess of BE or BA, which could explain their cytotoxicity [33,34].

To sum up, it must be realized that a cytotoxic compound in one cell line may well be much less active or even inactive in other cell lines, even within the same cancer type. The antiproliferative effect of the compound may also depend on the particular phase of the cell cycle. Therefore, there may be some differences in its properties. In our opinion, for precise measurements of cell viability (e.g., the cytotoxicity of tested compounds), it is worth comparing two or more different vitality tests, e.g., MTT and LDH with the ECIS method. It should be emphasized that the widely used MTT test (which allows for measuring the activity of energy transformations in mitochondria) should be used with caution, since many living cells may not show oxidative activity in mitochondria. Additionally, MTT was reported to interact with thiol-containing antioxidants [40], plant extracts [41], and other biologically relevant substances [42], leading to false positive endpoint measurements. Therefore, it is very important to select appropriate research methods to best capture the changes taking place in cells as a result of the action of the tested compounds, as well as to develop and implement them.

## 4. Material and Methods

### 4.1. Preparation of Betulin and Betulinic Acid

Both betulin and betulinic acid with purity >99% and rotenone with purity >95% were purchased from Sigma-Aldrich (St. Louis, MO, USA). Stock solutions of betulin (100 mM), betulinic acid (100 mM), and rotenone (1 mM) were prepared in DMSO (Sigma-Aldrich, St. Louis, MO, USA) and stored at −20 °C.

### 4.2. Cell Lines Cultures

Human lung carcinoma (A549) and human breast carcinoma (T47D) were obtained from the European Collection of Cell Cultures and were cultured in RPMI-1640 media, supplemented with 10% heat-inactivated fetal bovine serum (FBS, Gibco, Waltham, MA, USA), penicillin G (100 U/mL) (Sigma-Aldrich, St. Louis, MO, USA), and streptomycin (100 µg/mL) (Sigma-Aldrich, St. Louis, MO, USA). Cultures were kept at 37 °C in a humidified atmosphere of 95% air and 5% CO_2_. The control group was a Vero (fibroblast-like kidney from African green monkey) cell line obtained from Sigma-Aldrich and cultured in DMEM (Sigma-Aldrich, St. Louis, MO, USA), supplemented with L-glutamine (Gibco, Waltham, MA, USA) and 10% heat-inactivated fetal bovine serum (FBS, Gibco). All examined cell lines were tested against mycoplasma contamination with microbiological assays.

### 4.3. The Cell Proliferation Assay—MTT Assay

The cell viability was assessed by employing a 3-(4,5-dimethylthiazol-2-yl)-2,5-diphenyltetrazolium bromide (MTT) assay (Roche, Basel, Switzerland) in which the yellow tetrazolium salt was metabolized by viable cells to purple formazan crystals. T47D, A549, and Vero cells were seeded on 96-well microplates (Nunc) at the density of 1 × 10^4^ cells/well and left for 24 h. The next day, the culture medium was removed and the cells were exposed to serial dilutions (0, 1, 5, 10, 20, 40, 50, 75, and 100 µM) of BE and BA made in a serum-free medium, for 48 h. Additionally, the Vero cell line was exposed to rotenone in concentrations: 0.1, 1, 10, 100 nM, 1, and 10 µM. Each compound in each concentration was tested in triplicate. Next, the cells were incubated for 4 h with 20 µL of MTT solution (5 mg/mL). The formazan grains formed by viable cells were solubilized with 200 µL of DMSO, and the color intensity was measured at a 570 nm wavelength. The results were expressed as an IC_50_—the concentration of compound (in μM) that inhibits the proliferation rate of the tumor cells by 50%, as compared to the untreated control cells. For further experiments, values close to the IC_50_ concentrations of BE and BA were chosen for each line. The experiment was performed in three independent repetitions.

### 4.4. Cytotoxicity Assay—LDH Assay

A cytotoxicity detection kit based on the measurement of lactate dehydrogenase activity was applied (Tox-7, Sigma). The assay is based on the reduction of NAD by the action of lactate dehydrogenase released from damaged cells. The resulting NADH is utilized in the stoichiometric conversion of a tetrazolium dye. To evaluate the effect on normal cell viability, Vero cells were treated with increasing doses of BE and BA, ranging from 1 µM to 100 µM during a 48 h culture period. Next, cells were collected and incubated with substrate mixture for 30 min at room temperature, in the dark. In the end, the reaction was terminated by the addition of 1 N HCl, and the color product was quantified spectrophotometrically at a 450 nm wavelength.

### 4.5. Impedance Sensing Assay—ECIS Assay

The ECIS system’s Ztheta instrument (Applied Biophysics Ltd., Troy, NJ, USA) was used to measure the impedance. It contained two separate units: the station controller Zθ, located outside the incubator, and a docking station containing two 8-well plates, which were placed in the incubator space. The standard 8-well ECIS disposable arrays consist of gold film electrodes delineated with an insulating film and mounted on a 20 mil optically clear Lexan^®^ polycarbonate substrate. The eight-well top assembly is made of polystyrene. The gold layer is sufficiently thin (approx. 50 nm) to allow microscopic observation of the cells using a standard inverted tissue culture microscope. Each well has a surface area for cell attachment and growth of ~0.8 cm^2^ and holds a maximum volume of about 600 µL. Gold pads at the edge of the array connect electrodes to the ECIS electronics via contact with spring-loaded pins within the electrode array station. The electrodes used were 8W10E (Applied Biophysics Ltd., Troy, NJ, USA), which comprised 8 wells and 10 active electrodes in each well. ECIS electrodes were placed in a holder plate in a humid incubator at 37 °C and 5% CO_2_. Prior to inoculation, the arrays were incubated for 24 h with DMEM (Vero cells) and RPMI (A549, T47D cells) in the incubator overnight. Following stabilization, the array was removed from the array station and inoculated with cells. Inoculation of arrays was carried out by 600 µL/well of cell suspension ~1.2 × 10^5^ cells/mL. After 24 h, the examined compounds were added to inoculated wells in concentrations presented in Table 1, which were selected based on the MTT and LDH experiments’ results.

After cell manipulation, the matrix holder was placed in an incubator, and real-time measurements were initiated. The maximum response for Z, R, and C occurred at different frequencies. In this study, the default optimal frequencies were used: resistance (R) 4000 Hz, impedance (Z) 32,000 Hz and capacitance (C) 64,000 Hz. The changes in cellular behavior in response to the compound were recorded as impedance signals, and the data obtained were processed through ECIS software (Applied Biophysics Ltd., Troy, NJ, USA). After cell stimulation with the tested substance, the morphological changes that followed were expressed in the impedance values measured with the ECIS system.

## 5. Conclusions

Our study, with the use of lung (A549) cancer, breast (T47D) cancer, and normal epithelial cells (Vero), confirmed the possibility and justification of using the ECIS technique in anticancer in vitro research. Due to its non-destructive measurements, ECIS allowed for further cell testing after the end of the experiment. An unquestionable advantage is the live-tracking of changes in cell morphology, which reflects the processes of proliferation or cytotoxicity. Therefore, the ECIS method can be successfully used as a replacement or in combination with the commonly used colorimetric assays for cell viability testing, which could be translated to develop better treatment protocols and obtain the best possible cytotoxicity effect against cancer cells in time.

## Figures and Tables

**Table 1:** IC_50_ values of BE and BA on T47D and A549 cell lines as determined during 48 h period of MTT assay, using non-linear, four-parameter regression analysis.

	IC_50_ (µM)		
Compound/Cell Line	T47D	A549	Vero
BE	7.8	9.5	21.3
BA	5.4	6.9	18.6

## Data Availability

The data presented in this study are available on request from the corresponding author.
